# The Factorial Structure of Trait Anxiety and Its Mediating Effect Between Mindfulness and Depression

**DOI:** 10.3389/fpsyt.2018.00514

**Published:** 2018-10-26

**Authors:** Tao Wang, Min Li, Song Xu, Chenggang Jiang, Dong Gao, Tong Wu, Fang Lu, Botao Liu, Jia Wang

**Affiliations:** ^1^Department of Military Psychology, School of Psychology, Army Medical University, Chongqing, China; ^2^Maternal and Child Health Care Hospital, Chongqing, China; ^3^Daping Hospital, Army Medical University, Chongqing, China; ^4^School of Nursing, Army Medical University, Chongqing, China; ^5^School of Psychology, Army Medical University, Chongqing, China

**Keywords:** mindfulness, trait anxiety, depression, mindfulness-based cognitive therapy, personality

## Abstract

**Background:** Increasing studies have found that high trait anxiety is a key susceptibility phenotype that causes depression. Mindfulness-based interventions can target on dealing with depressogenic vulnerability effectively. Evidence indicates that trait anxiety could affect the trajectory of anti-depressive psychotherapy, and play an important role in the relationship between mindfulness and depression. Furthermore, related studies have found that trait anxiety could involve factors beyond anxiety and be a two-factor construct instead of one-dimensional concept. This viewpoint provides a new prospective for exploring the pathways of the two factors of trait anxiety in the complex relationship and further understand the potential mechanism of vulnerable personality mediated the link of mindfulness and depression.

**Methods:** A cross-sectional survey and a preliminary intervention study were conducted. Thousand two hundred and sixty-two subjects completed a set of self-reported questionnaires that evaluated trait anxiety, mindfulness, and depressive symptoms. Twenty-Three eligible participants with depression were recruited to attend mindfulness-based cognitive training for eight weeks. The same questionnaires were completed 1 week before the training and 6 months after the training. Factor analysis was performed on the 1262-subject sample to explore and confirm the factorial structure of trait anxiety. In addition, mediating effect analysis was conducted in the two studies to test whether two factors of trait anxiety were mediators of the relationship between mindfulness and depression.

**Results:** The exploratory factor analysis extracted two dimensions of trait anxiety, namely, trait anxiety-present factor (TA-P) and trait anxiety-absent factor (TA-A). And confirmatory factor analysis showed that the fit of the two-factor model was acceptable. Both TA-P and TA-A were significantly negatively correlated with mindfulness and positively correlated with depression, and they played a mediating role between mindfulness and depression. The two factors of trait anxiety had multiple mediating effects on the relationship between mindfulness and depression, and the mediating effect of the TA-P factor was stronger than that of the TA-A factor.

**Conclusion:** Our results demonstrated a two-factor model of trait anxiety in the Chinese population. TA-P and TA-A played a multiple mediating role in the relationship between mindfulness and depression. The findings provide new perspectives for psychological interventions to treat depression for people with susceptible personalities. Aiming to reduce negative emotional tendencies (TA-P factor) and enhance positive cognition (TA-A factor) may achieve the early prevention and efficient treatment of depression.

## Introduction

Depression is a common emotional disorder with complex and diversified symptoms. It has high rates of recurrence and shows trends of chronicity, and it sometimes leads to suicide. It is one of the diseases that the world health organization (WHO) focuses on (1). The diathesis-stress model of depression (2) suggests that individuals' susceptibility is closely related to the occurrence and development of depression (3) and may also affect the effectiveness and course of anti-depressive psychotherapy. In particular, studies have increasingly found that high trait anxiety is a key susceptibility phenotype that causes depression (4, 5). It has been found that individuals with high trait anxiety have characteristics such as dysregulation of attentional control (6) and cognitive biases (e.g., exaggerated threat scenarios) (7). They are more likely to adopt negative coping styles to address current dilemmas (8) and to have poor differentiation of negative affect (9). Moreover, their brain's functional connections are different from those of individuals with low trait anxiety (10).

In recent years, with the rise of mindfulness training, interventions based on mindfulness have been widely used in the prevention and treatment of depression, and they also have achieved very good therapeutic effects in reducing stress, and improving mood, mental health, quality of life and pain in clinical or non-clinical populations (11–13). Non-judgmental awareness, meditation, and acceptance are essential elements of mindfulness exercise (14, 15). Cognitive neuroscience studies have found that mindfulness can effectively change the activation pattern of brain regions associated with mood regulation (16, 17). Long-term mindfulness training can also lead to structural or morphological changes in the cortex thickness and gray matter density in brain regions associated with sensory processing, attention processes, and learning and memory (18, 19). Research have found that some special link exists between trait anxiety and meditation (20), and that trait anxiety is negatively correlated with mindfulness (21), mindfulness and meditation training can improve trait anxiety levels (22–24).

Previous studies demonstrated that the working effects of mindfulness-based intervention were mediated by alterations of mindfulness, self-compassion and cognitive reactivity across treatment (25) or improvement of cognitive functions (i.e., sustained attention, working memory) (26). Furthermore, some evidences suggested that dispositional characteristic (i.e., neuroticism) may involve the mechanism underlying the mindfulness and mental distress, and individuals in higher neuroticism can benefit more from mindfulness interventions (27). Considerable evidences demonstratethat high trait anxiety is particularly vulnerable to develop depression when facingstressful adversity (28). However, studies on the mediating effects and specific pathways of trait anxiety in the relationship between mindfulness and depression are limited.

As proposed by Spielberger (29), trait anxiety refers to the tendency of individuals to feel anxious and nervous while facing dangerous or uncertain scenarios. It is a relatively permanent characteristic. In the past, trait anxiety was considered a one-dimensional concept. However, in recent years, it has been found that trait anxiety may contain other factors beyond anxiety (30, 31). Scholars have proposed three different structural models:(a) the one-factor model of trait anxiety, where the 20 items in the Trait Anxiety Inventory are considered a common factor; (b) the two-factor model, in which all items contemporarily load on two uncorrelated factors (i.e., trait anxiety-present and trait anxiety-absent) and on a general factor (“negative affect”); and the (c) hierarchical model, which includes two lower-order factors with factors loading on a higher-order factor (“negative affect”). Which model can most accurately reflect the nature of trait anxiety? We use factor analysis methods to first identify possible structures of trait anxiety. Then, we further explore the influencing role of related structures of trait anxiety in the relationship of mindfulness and depression in cross-sectional and intervention studies, so as to delineate the pathways of trait anxiety sub-factors in the relationship.

Based on the above analysis, the hypotheses in this study are as follows: (a) Trait anxiety consists of a two-factor structure. (b) The two-factor structure of trait anxiety is closely related to mindfulness and depression. (c)Trait anxiety plays a mediating role in the relationship between mindfulness and depression, and (d) its possible paths include that mindfulness directly affects or predicts depression, and the association between mindfulness and depression is mediated indirectly by the two factors of trait anxiety.

## Materials and methods

### Participants

This study was approved by the ethics committee of Third Military Medical University of China (Document Number ChiCTR1800017168). And written informed consent was obtained after the procedures were clearly explained to the participants. The study included two parts. The first part was a cross-sectional investigation. Referring to the formula for large sample population (32), N≥($\frac{k}{a}$)^2^*P*(1-*P*), we defined the values of the formula as alpha of significance level (a) = 0.05, its value for 95% confidence level (k) = 1.96, *p* = 0.5, and the sample size was 385. At the same time, 15% of no response rate was considered and the minimum number of subjects required for the study was at least 443. Our final valid sample contained 1,262 subjects, 74.72% of them were female, average age was 33.29 years (range: 19–60 years).They were all working people of Han nationality from central city in China. 70.36% of the respondents are married, while the rest of the sample are categorized into single (27.26%) and divorced (2.38%). 54.96% of the participants receive education beyond undergraduate level, and the others are beyond the middle school.

The second part was a preliminary experiment. Subjects with depressive symptoms were recruited online or at psychological clinic. A total of 23 eligible volunteers took part in the training, including 7 males and 16 females. The age range was 25–44 years (mean:33.43, SD:6.13).Their educational background is beyond middle school level. The majority of them are employed except one. Among them, 56.52% of participants are married, 34.78% are single, and 8.70% are divorced. The duration of depressive symptom is from 2 months to 13 years. Under the guidance of a psychiatrist, five participants with a history of antidepressant medication stopped taking or maintained with low dosage for at least 1 month before the training, and others have no use of medication.

## Procedure

The subjects of the cross-sectional study were from public institutions in two different cities in China. To ensure the validity of the sample, all subjects were tested and treated by psychologists on site. All results of psychological questionnaires were reported to the subjects through e-mail.

In the intervention experiment, recruitment information about the research purpose, content, and method, along with a participation form, was first published through paper and online posters. Then, structured interviews were conducted by a psychiatrist, a clinical psychologist, and the investigator. Twenty-three individuals with depressive symptoms underwent psychological assessments one week before the training and six months after the training.

The inclusion criteria and exclusion criteria for subject recruitment are as follows.

Inclusion criteria: (a) Hamilton depression scale (HAMD-24) (33) score > 8 points, which was simultaneously assessed by one clinical psychiatrist, one psychologist and the first author; (b) Beck Depression Inventory (BDI) (34, 35) score ≥14 points; (c) age: 18~50 years old; and (d) education level of junior high school level or above.

Exclusion criteria: (a) experiencing an acute episode of depressive disorder; (b) having bipolar affective disorder; (c) having other major mental diseases (such as schizophrenia, personality disorder) or cardiovascular and other physical diseases; and (d) having an education level below the junior high school level and being unable to understand the content of the scale.

Eight mindfulness-based cognitive training (MBCT) sessions were conducted once a week for 2 to 2.30 h each session. MBCT was a structured psychological training program for addressing depressogenic vulnerability. Participants were taught to experience thoughts, bodily sensations and emotions in a non-judgmental and compassionate way, thereby learn to disengage from dysfunctionally cognitive and emotional processes (14, 36). In our MBCT training, weeks one to four were for basic mindfulness exercises (the body scan, meditation, mindfulness movement, mindfulness of breathing), which highlighted to be aware of automatic habitual patterns of reactivity (e.g., negative thoughts and unpleasant feelings) during meditation. And the next 4 weeks were focused on emotional changes through the following exercises and activities: letting go of judgment, decentering from negative thought streams, addressing barriers, and making a plan to better care for oneself. In addition to these group exercises, the participants also were required to commitment to daily practice with the aid of guided MP3 recordings for optimal benefit. The twenty-three participants were trained in mindfulness in two batches (10 in the first batch and 13 in the second batch).The training was guided by 1 to 2 mentors with mindfulness training.

## Measures

### Trait anxiety subscale

The Chinese version (37) of the Trait Anxiety Scale was revised based on the State-Trait Anxiety Inventory, Trait Version, Form Y (STAI-T) (29). The scale had 20 items that were scored from 1 to 4 (1 = completely absent, 4 = very obvious) and described a relatively stable personality trait and anxious tendencies. It was used to assess people's regular emotional experience. Some items were reversely scored. The trait scale had a Cronbach's alpha coefficient of 0.869 in the cross-sectional study and 0.819 in the experimental study.

### Beck depression inventory (BDI)

The Beck Depression Inventory (35) was used to assess the severity of depressive symptoms. It has 21 items, each containing 4 statements, with Arabic numerals marked before each statement to indicate scores from 0 to 3. A total score of 14 points or more is considered to indicate a mild or high depression levels. Cronbach's alpha for BDI in the cross-sectional and experimental studies was 0.93 and 0.817, respectively.

### Five facet mindfulness questionnaire (FFMQ)

The Five Facet Mindfulness Questionnaire (38) contains a total of 39 items that are scored from 1 (fully non-consistent) to 5 (fully consistent). It includes 5 dimensions: mindfulness observations, mindfulness descriptions, awareness actions, no judgments, and no responses. The FFMQ score was obtained by adding up the scores on the five dimensions. The higher the score, the higher the level of mindfulness. In this study, Cronbach's alpha coefficients for the five subscales ranged from 0.674 to 0.847.

### Variables and statistical methods

All data were processed using SPSS19.0 and AMOS24.0 software. Descriptive statistics were used to analyze the mean and standard deviation of the observed variables. Pearson's correlation analysis was used to evaluate the relationships among the observed variables and to test the reliability of the questionnaire. The paired *t*-test was used to analyze the effect of MBCT training on depression. Differences were considered significant at *p* < 0.05.

To clarify the structure of trait anxiety, the whole sample in the cross-sectional study was randomly divided into half. Half of the data (*n* = 622) were analyzed by SPSS19.0 to identify the exploratory factors of the Trait Anxiety Scale, and the other half (*n* = 620) were analyzed by AMOS 24.0 to examine the stability of the two-factor solution derived from exploratory factor analyses. Subsequently, structural equation modeling (SEM) was used to analyze the mediating effect of trait anxiety between mindfulness and depression and to test the significance of the mediating effect. The hypothesized mediation model was estimated using SEM in AMOS 24.0 in both the cross-sectional study and the experimental study. The overall model fit was evaluated with the following indices: CMIN/*df* (the minimum value of sample discrepancy divided by its degree of freedom, with less than five indicating an acceptable model fit); the root mean square error of approximation (RMSEA), with a value less than 0.08 indicating an adequate fit; and the incremental fit index (IFI), the comparison fit index (CFI), the goodness-of-fit index (GFI), and the adjusted-goodness-of-fit index (AGFI), where a value greater than 0.90 is preferable. In addition, the bootstrapping method was performed to test the significance of the mediation effect.

## Results

### Factor analysis

In the first part of the sample (*n* = 622), exploratory factor analysis (EFA) was performed using 20 items contained in the scale as analysis indicators. The results showed that the Kaiser-Meyer-Olkin (KMO) value was 0.919, and Bartlett's test of sphericity reached a level of significance(*P* < 0.001), indicating that was suitable for factor analysis. Principal factor analysis and varimax rotation method were used to extract the initial factors. Two factors were identified with eigenvalues > 1, accounting for 51.22% of the total variance. One factor included 9 items (items 1, 3, 6, 7, 10, 13, 14, 16, 19), explaining 27.74% of the variance, and another factor contained 11 items (items 2, 4, 5, 8, 9, 10, 11, 12, 15, 17, 18, 20), explaining 22.48% of the variance. The extracted two-factor structure was roughly equivalent to the structure proposed by previous related research (31). The factor contained 9 items that were reversed scored on the scale, and all items represented positive emotions. In another factor, 10 items reflected positive emotional experiences, except for item 4, which reflected negative emotional experiences. The component loadings of most items in the two factors were 0.589~0.812, except for item 4 (−0.467). To faithfully reflect the psychometric properties of the original scale and avoid conceptual ambiguity, we used the Trait Anxiety Scale to label the two factors TA-P factor and TA-A factor.

To examine the stability of the two-factor structure of trait anxiety derived from EFA, we performed a confirmatory factor analysis (CFA) on the other half of the data (*n* = 620), and the maximum likelihood method was used to examine the fit index of the three models. The CFA results indicated that model 1 showed a poor fit to the data (i.e., 0.158 for RMSEA), and model 2 indicated an acceptable fit, with all fit indices either “good” (CFI = 0.903, IFI = 0.904) or “fair” (RMSEA = 0.071, GFI = 0.898). Furthermore, the standardized factor loading of each item on the factor was between 0.56 and 0.78 (except item 4, whose standardized factor loading was −0.31). Cultural differences in the comprehension of this item could be one possible interpretation of why item 4 had a low item-factor correlation. The content of this item is “I wish I could be as happy as others seem to be.” Model 3 could not be identified. The goodness-of-fit indicators for the three models are shown in Table 1.

**Table 1:** Fit indices of hypothesized models of trait anxiety inventory (*n* = 620).

**Model**	***χ^2^***	***df***	****X**^2^/*df***	**RMSEA**	**NFI**	**CFI**	**IFI**	**RMR**	**GFI**	**AGFI**
Model 1	2849.09	170	16.76	0.158	0.504	0.518	0.519	0.104	0.491	0.372
Model 2	707.15	170	4.16	0.071	0.877	0.903	0.904	0.065	0.898	0.874
Model 3	The model is unidentified.									

*Model 1, one-factor model of trait anxiety; Model 2, the two-factor model of trait anxiety; Model 3, the hierarchical model*.

### The reliability and validity tests

The reliability of the Trait Anxiety Scale was tested. The results showed that the internal consistency coefficients (Cronbach's alpha) of the total scale, TA-P factor, and TA-A factor were 0.869, 0.899, and 0.821, respectively. Their split-half reliability was 0.864, 0.858, and 0.819, respectively. With regard to the validity of the scale, there was a strong positive correlation between TA-P factor and-TA-A factor and the total score of trait anxiety (*r*_TA_-_P_ = 0.838, *r*_TA_*-*_A_ = 0.762, *P* < 0.001), and the two factors were weakly related (*r* = 0.284, *P* < 0.001). This shows that the scale had good structural validity, and both of the measured factors had a common construct and their own independent roles.

### Descriptive statistics and correlation analysis of variables in cross-sectional study

Spearman's correlation analysis found that mindfulness was significantly negatively correlated with the TA-P factor, the TA-A factor, and BDI and was moderately positively correlated with the TA-P factor and BDI (*p* < 0.001). There was a low positive correlation between TA-A factor and TA-P factor (*p* < 0.001). The descriptive statistics and correlation analysis of each observation variable are shown in Table 2.

**Table 2:** Descriptive statistics and correlation analysis of variables in cross-sectional study.

	**Range**	**Mean**	**SD**	**1**	**2**	**3**	**4**
1.FFMQ	80~156	118.56	9.14	1		
2.TA-P factor	11~41	19.00	4.83	−0.352^***^	1	
3.TA-A factor	9~36	23.09	5.93	−0.404^***^	0.239^***^	1
4.BDI	0~47	8.81	9.41	−0.249^***^	0.539^***^	0.352^***^	1

*** *p < 0.001. FFMQ, Five Facet Mindfulness Questionnaire; TA-P factor, trait anxiety-present factor; TA-A factor, trait anxiety-absent factor; BDI, Beck Depression Inventory; SD, standard deviation*.

### Paired *t*-tests for each variable before and after mindfulness training

Paired *t*-tests were performed to compare the scores of the observed variables before and after mindfulness training. The results showed that the level of mindfulness was significantly improved after training, the TA-P factor and A factor scores of trait anxiety were significantly reduced, and depression severity was significantly reduced. The results are shown in Table 3.

**Table 3:** Paired *t*-test of observed variables before and after mindfulness training ($\bar{\chi }$ ± s).

	**One week before training**	**Six months after training**	***d* value**	***t***
FFMQ	102.70 ± 12.01	120.13 ± 17.44	−17.43	−5.84^***^
TA-P factor	27.91 ± 7.26	20.65 ± 5.86	7.26	4.72^***^
TA-A factor	31.00 ± 5.33	22.79 ± 4.69	8.22	7.53^***^
BDI	26.04 ± 10.18	8.52 ± 10.15	17.52	7.57^***^

*** *P < 0.001. FFMQ, Five Facet Mindfulness Questionnaire; TA-P factor, trait anxiety-present factor; TA-A factor, trait anxiety-absent factor; BDI, Beck Depression Inventory*.

### Mediating effect analysis of two factors of trait anxiety

**Figure 1:**
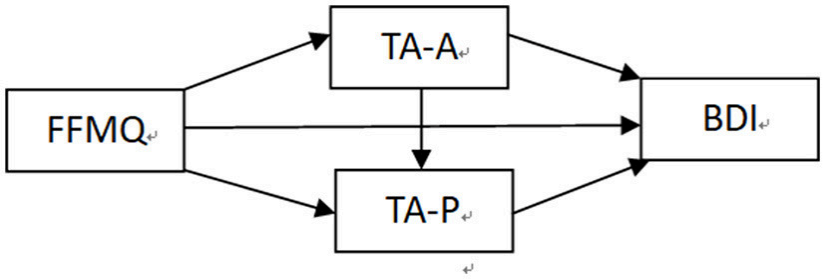
Hypothetical model of trait anxiety. FFMQ, Five Facet Mindfulness Questionnaire; TA-P factor, trait anxiety-present factor; TA-A factor, trait anxiety-absent factor; BDI, Beck Depression Inventory.

Regression analyses were performed to evaluate the direct effect of dependent variable (BDI score) on FFMQ in the cross-sectional data (*n* = 1262). The demographic characteristics (gender, age) were included as a covariate, while the FFMQ was entered as an independent variable. The results showed that the direct effect from FFMQ to the BDI was significant (β = −0.248, *SE* = 0.029, *t* = −8.650, *p* < 0.001). Based on the literature review and the aforementioned data, we proposed a hypothesis model for the relationship between TA-A and TA-P for mindfulness and depression, as shown in Figure 1. The mediation model was set up as shown in Figure 1. First, the model was established as saturated model, and SEM was used to estimate the model fit. The direct path from FFMQ to BDI was insignificant (β = −0.022, *SE* = 0.027, *t* = −0.817, *p* > 0.05). After the insignificant direct path in the saturated model was deleted, the modified model was obtained with an acceptable goodness-of-fit-index(χ^2^ = 0.668, *df* = 1, χ^2^/*df* = 0.668, RMSEA = 0, CFI = 1.000, TLI = 1.002, SRMR = 0.005).

In addition, the bias-corrected bootstrap method proposed by Preacher and Hayes (39) was used to test the statistical significance of the mediating effects. Two thousand repeated samplings were taken from the data set, and the 95% confidence interval (excluding 0) was statistically significant. The results showed that the 95% CI of all pathways did not include 0, indicating a significant mediating effect, as shown in Table 4. After we controlled for the mediator variables, the impact of mindfulness on depression diminished (β = 0.020, SE = 0.027, *t* = 0.745, *p* > 0.05, 95% CI [−0.03, 0.074]), and the overall indirect effect of the 95% CI did not contain 0 (−0.313, −0.2283); thus, it played a full mediating role.

**Table 4:** Two factors of trait anxiety as mediators between FFMQ and BDI in cross-sectional study (*n* = 1262).

**Path**	**Standardized indirect effect estimation**	**Average indirect effect**	**95% CI**	
			**Lower**	**Upper**
Path 1: FFMQ—TA-A—BDI	(−0.36) × 0.24 = −0.086	−0.102	−0.127	−0.078
Path 2: FFMQ—TA-P—BDI	(−0.35) × 0.48 = −0.168	−0.142	−0.176	−0.109
Path 3: FFMQ—TA-A—TA-P—BDI	(−0.35) × 0.11 × 0.24 = −0.009	−0.025	−0.039	−0.012

*FFMQ, Five Facet Mindfulness Questionnaire; TA-P factor, trait anxiety-present factor; TA-A factor, trait anxiety-absent factor; BDI, Beck Depression Inventory;95% CI, 95% confidence interval*.

Mediating effect analysis was also performed on the 23 subjects receiving 8 weeks of mindfulness training. The analysis procedure was consistent with that of the cross-sectional study. The difference was that all variables were *d* values of the observed variables before and after the intervention. The results showed that the direct path between FFMQ-BDI was still insignificant (χ^2^ = 0.085,*df* = 1, *X*^2^/*df* = 0.085, RMSEA = 0, CFI = 1.000, TLI = 1.119, SRMR = 0.0108, NFI = 0.997, RFI = 0.985). While path 3 contained0, the 95% CI of paths 1 and 2 did not contain 0, indicating that the mediating effect of these two paths was significant, as shown in Table 5. After controlling for mediating variables, the impact of mindfulness on depression became insignificant (β = 0.044, SE = 0.167, *t* = 0.266, *p* > 0.05, 95% CI [−0.307, 0.396]), and the overall indirect effect 95% CI did not contain 0 [−0.729, −0.117]. It played a full mediating role.

**Table 5:** Mediating effect of trait anxiety in the preliminary experimental study (*n* = 23).

**Path**	**Standardized indirect effect estimation**	**Average indirect effect**	**95%CI**	
			**Lower**	**Upper**
Path 1: FFMQ_d_–TA-A_d_–BDI_d_	(−0.36) × 0.37 = −0.168	−0.123	−0.435	−0.005
Path 2: FFMQ_d_–TA-P_d_–BDI_d_	(−0.49) × 0.45 = −0.221	−0.127	−0.382	−0.049
Path 3: FFMQ_d_–TA-A_d_–TA-P_d_–BDI_d_	(−0.36) × 0.35 × 0.45 = −0.057	−0.061	−0.272	0.011

*FFMQ_d_, d value of Five Facet Mindfulness Questionnaire; TA-P_d_ factor, d value of trait anxiety-present factor; TA-A_d_ factor, d value of trait anxiety-absent factor; BDI_d_, d value of Beck Depression Inventory; 95%CI: 95% confidence interval*.

## Discussion

The exploratory factor analysis extracted two dimensions of trait anxiety in the present study. The confirmatory factor analysis showed that the fit index of the two-factor structure model was acceptable. It indicated that the first-order factors of trait anxiety were established, but the second-order factors could not be further fitted. One possible interpretation is that there was a low correlation between the two factors of trait anxiety, and only has two first-order factors in the model which do not meet the criteria of at least three first-order factors (40).

Regarding the dimension of trait anxiety, even Spielberger himself (30) thought that some items reflected depression-related emotions, such as dysphoric mood and self-deprecation. Some scholars have also found that the contents of the scale contained a considerable number of general negative emotions, such as tension, anxiety, frustration, and jealousy; they stated that the scale was not a tool to assess anxiety in a pure sense and may instead reflect a non-specific worry or contemplation process (30, 41, 42). In other words, the trait anxiety questionnaire may assess a general, non-specific susceptibility tendency that individuals present in the process of information processing, including two complex but different dimensions of negative emotions (31, 43). Our study obtains similar results and concludes that the Trait Anxiety Scale assesses negative emotion tendency and contains two factorial structures: The TA-A factor consists of nine reversed-scored items and reflects the positive emotional experience of regular enjoyment, quietness, happiness, self-satisfaction, etc. Meanwhile, all ten positively scored items and the reverse-scored item 4 form the TA-P factor, which investigates regular negative emotional experiences such as nervousness, anxiety, and depression.

The study found that factors TA-P and TA-A were significantly negatively correlated with mindfulness and positively correlated with BDI. Before and after the mindfulness training, in addition to the significant decrease in the BDI score, the scores of the two factors of trait anxiety were also significantly reduced, and the level of mindfulness was obviously improved. Structural equation modeling was used to evaluate the mediating effect. It was found that in the two groups of different populations and different study designs, the adjusted mediation effects were significant. This indicates that TA-P and A factor play a mediating role between mindfulness and depression and that the two-factor structure of trait anxiety can have multiple mediation effects on the impact of mindfulness on depression. The mediating effect of trait anxiety P factor was the most obvious, accounting for the largest proportion in the two different studies (63.88 and 71.20%), whereas the mediating effect of factor A was significantly lower (32.70 and28.8%). Interestingly, in the cross-sectional data, mindfulness also influenced the level of depression by affecting the A-P factor chain. Although the mediating effect was relatively low and accounted for only 3.42%, this chain-mediated effect in the preliminary study was not obvious. Therefore, the improvement in depression by mindfulness is more strongly affected by the relieving of P factors (negative emotions) in trait anxiety, and the effect of the A factor in influencing depression was clearly smaller.

Both datasets found that the direct effect of mindfulness on depression became statistically insignificant after the two-factor variable of trait anxiety was controlled for, and the two factors of trait anxiety played a complete mediation role. With regard to full and partial mediation, some scholars believe that the existence of mediating effects should be based on whether the direct effect c′ is statistically significant and that the existence of mediating effects should be a matter of how large the mediating effect is; its dichotomous existence should not be a concern (44). Additionally, it is believed that the proposal of the concept of complete mediation excludes the possibility of exploring other intermediaries in the future (45) and does not conform to the reality of the study of complex psychological phenomena. Therefore, Preacher and Hayes (39) called for abandoning complete mediation and taking all mediations as partial mediations. Our study finds that trait anxiety personality has a significant mediating effect between mindfulness and depression. This result confirms the important role of the two factors of trait anxiety in the relationship between mindfulness and depression, thus providing us with a new perspective for better understanding the mechanism between mindfulness and depression.

Spielberger et al. studied the trait anxiety personality dimension and provided us with a new direction for further exploring the personality basis of emotional disorder. However, with the deepening of research, the factorial structure of trait anxiety has become a new topic. We find that the Trait Anxiety Inventory contains TA-P and TA-A dimensions. The P factor is related to moderate depression, while the A factor is slightly related to depression. The results support the two-factor model proposed in the research on trait anxiety.

The current research on trait anxiety mainly focuses on the following aspects: First, because anxiety and depression are highly relevant, scholars have long sought to find a measurement tool that can effectively distinguish between anxiety and depression. It is believed that there is a need to further improve or revise the content of items. For example, to reduce items that overlap with depression and increase the physiological arousal of anxiety to more purely evaluate the important psychological concept of trait anxiety (31, 41). Second, scholars have explored the dimensions of existing scales and proposed new alternative concepts, such as the concept of negative affectivity in the tripartite model of depression and anxiety (46). Some scholars believe that trait anxiety reflects a general negative emotion, which is a common element of anxiety and depression (43). In fact, Spielberger also proposed the concept of trait depression (41, 47), which is mainly used to investigate the frequency of individual negative emotions and positive emotions. If trait anxiety is more closely related to negative emotions, what is the relationship with trait depression? The effectiveness in distinguishing between anxiety and depression remains to be further studied. Third, the study attempted to determine the differences between different individuals with trait anxiety in the cognitive neural processing and their possible neurobiological mechanisms (48, 49) to provide empirical evidence for improved or innovative treatment methods. Related studies have found that individuals with high trait anxiety have significant differences in cognitive processing, behavioral responses, and physiological responses attributed to different genes, molecules, brain functions, and structures (4, 50). The past view held that personality factors were relatively stable and difficult to change, but dynamic models of personality-depression relations (51) suggest that although early susceptibility forms a baseline level of individual disease risk, later experiences can modify and adjust the susceptibility of personality to depression, and positive life experiences can prevent or even reverse the pathological development path of personality.

Mindfulness aims to promote physical and mental health by guiding individuals to fully perceive and accept the experience of all kinds of cognition, thoughts, emotions, and physical changes that are currently occurring without judging. In recent years, studies have found that mindfulness has a positive influence on personality (52), focusing on the improvement and promotion of personality to achieve effective intervention for certain addictive disorders (53). Our study also finds that mindfulness promotes depression by improving trait anxiety levels, suggesting that the healing mechanism for depression in mindfulness is related to changes in trait anxiety. In addition, our research further explores the relationship between trait anxiety factors P and A in affecting mindfulness and depression in the form of multiple mediations, and each path has a different effect size. Research findings have revealed another key factor and important path for preventing and treating depression with mindfulness. However, because trait anxiety itself reflects tendencies in cognitive and emotional styles, more studies are needed to confirm whether EPQ, NEO, or neurotic personality in the Big Five personality dimensions or other personality dimensions also play a similar role between mindfulness and depression.

The mindfulness revolution continues to ascend. Interventions related to mindfulness and explorations of its mechanism for improving depression continue to develop. In our study, we propose the multiple mediating mechanism between the two factors of trait anxiety in mindfulness and depression from the perspective of susceptible personality. The study of the relationship between mindfulness and depression provides new perspectives and ideas. Depression can be improved by focusing on reducing individuals' negative emotional tendencies and enhancing positive cognition.

This study also has some limitations. First, the cross-sectional study used a cluster sampling method, and the representative of study population was limited. Future studies should strengthen the stratified sampling in terms of gender and age. Second, considering that the improvement in personality usually does not happen in a short period (51), the observing time was set to 6 months after MBCT training in our preliminary study. But this led the missing of some important information. Therefore, measurement at distinct time points over a long period of time(e.g., after training, 1 month later, 3 months later, 1 year later) may help us to collect more valuable information on the observed indicators and dynamically observe the change trend of personality and depression. Third, the lack of non-intervention control group in the experimental study made the results insufficient to verify the interventional effects of mindfulness training. The overall sample size was too small and should be increased to further validate the research results. For the reason, the generalization of the current findings should be made with caution, and more research in these areas is needed. Fourth, our current results still need to be further tested in patients with clinical anxiety and depression.

Despite these limitations, our study finds that trait anxiety consists of two factors, TA-P factor and TA-A factor. These two factors are correlated moderately with mindfulness and depression, and they play important multiple mediating roles between mindfulness and depression. In the future, the prevention and treatment of depression can focus on individuals' susceptibility levels, reduce negative emotional orientation through intervention or training, and promote positive cognition to achieve early prevention and treatment of depression.

## Author contributions

TaW, ML, and SX designed the research. TaW, ML, SX, CJ, and DG were involved in recruiting the participants and conducting the assessments. TaW, ML, and SX led MBCT training. ToW, FL, BL, and JW analyzed the data. TaW, ML, CJ, and SX wrote and revised the paper.

### Conflict of interest statement

The authors declare that the research was conducted in the absence of any commercial or financial relationships that could be construed as a potential conflict of interest.
